# Vitrification of Dog Skin Tissue as a Source of Mesenchymal Stem Cells

**DOI:** 10.1155/2021/1340281

**Published:** 2021-07-10

**Authors:** Young-Bum Son, Yeon Ik Jeong, Sang-Yun Lee, Yeon Woo Jeong, Ki-June Lee, Woo Suk Hwang

**Affiliations:** ^1^Abu Dhabi Biotech Research Foundation, 64 Kyungin-ro, Guro-gu, Seoul, Republic of Korea; ^2^Department of Theriogenology and Biotechnology, College of Veterinary Medicine and Research Institute of Life Science, Gyeongsang National University, Jinju, Republic of Korea

## Abstract

The purpose of this study was to develop an efficient vitrification system for cryopreservation of dog skin tissues as a source of stable autologous stem cells. In this study, we performed vitrification using four different cryoprotectants, namely, ethylene glycol (EG), dimethyl-sulfoxide (Me2SO), EG plus Me2SO, and EG plus Me2SO plus sucrose, and analyzed the behaviors of cells established from warmed tissues. Tissues vitrified with 15% EG, 15% Me2SO, and 0.5 M sucrose had a normal histological appearance and the highest cell viability after cell isolation, and thus, this cocktail of cryoprotectants was used in subsequent experiments. We evaluated proliferation and apoptosis of cells derived from fresh and vitrified tissues. These cells had a normal spindle-like morphology after homogenization through subculture. Dog dermal skin stem cells (dDSSCs) derived from fresh and vitrified tissues had similar proliferation capacities, and similar percentages of these cells were positive for mesenchymal stem cell markers at passage 3. The percentage of apoptotic cell did not differ between dDSSCs derived from fresh and vitrified tissues. Real-time PCR analysis revealed that dDSSCs at passage 3 derived from fresh and vitrified tissues had similar expression levels of pluripotency (OCT4, SOX2, and NANOG), proapoptotic (BAX), and antiapoptotic (BCL2 and BIRC5) genes. Both types of dDSSCs successfully differentiated into the mesenchymal lineage (adipocytes and osteocytes) under specific conditions, and their differentiation potentials did not significantly differ. Furthermore, the mitochondrial membrane potential of dDSSCs derived from vitrified tissues was comparable with that of dDSSCs derived from fresh tissues. We conclude that vitrification of dog skin tissues using cocktail solution in combination of 15% EG, 15% Me2SO, and 0.5 M sucrose allows efficient banking of these tissues for regenerative stem cell therapy and conservation of genetic resources.

## 1. Introduction

Regenerative medicine using stem cells focuses on restoring organs and cells that fail to function properly due to accidents or degenerative disease. Autologous stem cells are favored for effective disease treatment and prevent an immune reaction [1–3]. However, the establishment and culture of stem cells should be performed in a laboratory, and it is difficult to acquire high-quality cells in the case of illness or sudden accidental death. Therefore, there is a need to develop a tissue cryopreservation technique capable of maintaining the characteristics of stem cells.

Tissues can be cryopreserved by slow-freezing and vitrification techniques [4, 5]. The slow-freezing method is generally used for tissue cryopreservation [6, 7]. However, it requires specialized equipment for programmed freezing, has a long freezing time of more than several hours, and is hampered by ice formation [8]. These problems seem to be insurmountable, and therefore, attention has shifted to a simpler approach and the development of vitrification technology, which is widely used in cryobiology [8, 9]. Vitrification has a relatively short freezing time, is inexpensive, is not associated with extracellular or intracellular ice formation, and can be performed anywhere using only liquid nitrogen [10, 11]. Various tissue vitrification protocols have been reported using the intracellular cryoprotectants dimethyl-sulfoxide (Me2SO), ethylene glycol (EG), and glycerol as well as the extracellular cryoprotectants sucrose, fetal bovine serum (FBS), trehalose, and raffinose [12, 13]. However, vitrification requires a high cryoprotectant concentration, meaning problems such as cytotoxicity and excessive cryoprotectant penetration of the cell membrane should be addressed [14, 15]. Extracellular cryoprotectants affect viscosity and promote glass formation to reduce toxicity, and therefore, low concentrations of intracellular cryoprotectants can be used without impairing vitrification [16, 17]. Accordingly, studies have used extracellular cryoprotectants together with intracellular cryoprotectants [16, 17]. Therefore, in this study, we focused on the development of an efficient vitrification method for tissues, which are a potential source of stem cells, using a cocktail of cryoprotectants. Animal serum proteins and albumin increase the efficiency of vitrification and reduce cryoinjury [18]. However, they can be contaminated by infectious agents and change the characteristics of cells [19]. Therefore, the development of a cryopreservation method without xenogeneic animal serum, including FBS, can increase the utility of stem cells in regenerative medicine [20].

Tissue cryopreservation can be used to obtain autologous cells of patients for clinical applications and to preserve animal genetic resources [21]. Various tissues have been studied as sources of stem cells. Among them, skin tissue is a good candidate for regenerative medicine because of its excellent accessibility and availability with minimally invasive procedures. Skin tissue can be preserved without losing multipotency and is a source of autologous stem cells for regenerative medicine. However, there are very few reports regarding which cryoprotectants are suitable for dog skin tissue vitrification. Furthermore, most studies of skin tissue vitrification are limited to the establishment of unspecified cell lines and assessment of their survival, and studies of the establishment of mesenchymal stem cells (MSCs) for regenerative medicine and analysis of their characteristics are insufficient [22–24]. Therefore, in the present study, we aimed to standardize the cryopreservation methods for canine skin tissues as a source of autologous stem cells. The optimal vitrification method used a modified cocktail of cryoprotectants, which was previously used to vitrify mammalian ovarian tissues [25, 26].

## 2. Materials and Methods

All chemicals used in this study were purchased from Sigma (St. Louis, MO, USA) unless otherwise mentioned. All media were adjusted to pH 7.4 and an osmolality of 280 mOsm/kg, except for cryoprotective media and media used to wash warmed samples.

### 2.1. Animals

All animal studies were performed according to the animal study guidelines which were approved by the ethics committee of the Abu Dhabi Biotech Research Foundation, Korea (Permit no. C-20-01). These guidelines comply with the ARRIVE guidelines and are in accordance with the UK Animals (Scientific Procedure) Act 1986 and associated guidelines and EU Directive 2010/63/EU.

### 2.2. Vitrification of Dog Skin Tissues

Skin samples were obtained from the inguinal region of six mixed breed dogs (three males and three females) with an average age of 1 year. Dog skin tissues were washed with Dulbecco's phosphate-buffered saline (DPBS) containing 1% antibiotic-antimycotic solution, cut into 1 mm^2^ explants at 25°C, and divided into the following six groups: fresh (fresh group), vitrification with DPBS (DPBS group), vitrification with 40% EG (40% EG group), vitrification with 40% Me2SO (40% Me2SO group), vitrification with 20% EG and 20% Me2SO (20%EG + 20%Me2SO group), and vitrification with 15% EG, 15% Me2SO, and 0.5 M sucrose (15%EG + 15%Me2SO + 0.5 M sucrose group). All cryoprotectants were diluted using DPBS. In the vitrification process, minced skin tissues from single donors were exposed to the preequilibration solution for 1 min and transferred to 1 ml DPBS and cryoprotectant solutions in cryovials (Nunc, Roskilde, Denmark). The cryovials were subsequently plunged into liquid nitrogen. Detailed information about the preequilibration and cryoprotectant cocktail solutions is provided in Table 1. We stored vitrified dog skin tissues for three weeks.

### 2.3. Histological Assessment

After 3 weeks of tissue cryopreservation, tissues were warmed as described below and fixed with 10% formalin at room temperature for 24 h. Thereafter, the skin tissues were dehydrated using graded ethanol and embedded in paraffin. The skin tissue sections were deparaffinized with xylene and rehydrated. The slices were stained with hematoxylin and eosin (H&E) and then washed with water. The tissue sections were observed using a light microscope.

### 2.4. Isolation and Culture of Dog Skin Stem Cells from Fresh and Vitrified Tissues

The warming procedure of cryovials was performed as previously reported with minor modifications [13]. During the warming process to remove cryoprotectants, vitrified skin tissues were kept at room temperature for 5 min. They were incubated in Dulbecco's modified Eagle's medium (DMEM; Thermo Fisher Scientific, Waltham, MA, USA) supplemented with 0.3 M sucrose and 10% FBS (Invitrogen, Carlsbad, CA, USA) for 5 min at 38°C followed by DMEM supplemented with 0.15 M sucrose and 10% FBS for 5 min. Dog dermal skin stem cells (dDSSCs) were isolated from fresh and vitrified skin tissues as previously reported with a minor modification [20]. In brief, dermal skin tissues were minced with a surgical blade and incubated in DMEM containing 1 mg/ml collagenase type I (Thermo Fisher Scientific, Waltham, MA, USA) at 39°C in a humidified incubator containing 5% CO_2_ and 5% O_2_ with gentle agitation for 2 h. The digested tissues were washed twice with DMEM containing 10% FBS by centrifugation at 300 × g for 5 min. The fragments were filtered through 100 and 40 *μ*m nylon cell strainers (Falcon®, Franklin, NJ, USA) to obtain single-cell suspensions. After filtering, cells (3 × 10^5^) were cultured in 35 mm plastic culture dishes with DMEM containing 10% FBS, 1% (*v*/*v*) nonessential amino acids (Invitrogen), 1% antibiotic-antimycotic solution, and 0.1% *β*-mercaptoethanol (Thermo Fisher Scientific) at 39°C in a humidified atmosphere containing 5% CO_2_ and 5% O_2_. The culture media was changed every 2 days until confluency reached 80%, and then, cells were passaged.

### 2.5. Determination of the Survival Rates of dDSSCs Derived from Fresh and Vitrified Tissues

After being isolated from fresh and warmed vitrified tissues, 3 × 10^5^ dDSSCs were cultured in 6-well plates (Nunc, NY, USA). Following attachment, at 48 h after warming, cells were stained with propidium iodide (PI) to label dead cells and with Hoechst 33342 to label all cells as previously reported [20, 27]. Stained single cells were observed using a fluorescence microscope (Nikon Eclipse Ti-U; Nikon Instruments, Tokyo, Japan), and the cell survival rate was calculated in each group as previously reported [20, 27]. Based on these results, dDSSCs derived from fresh tissues (fresh group) and tissues vitrified using 15% EG, 15% Me2SO, and 0.5 M sucrose (cryogroup) were used in further experiments.

### 2.6. Analysis of Cell Proliferation and Apoptosis

To evaluate cell proliferation, dDSSCs in the fresh and cryogroups were seeded at a density of 2 × 10^3^ cells per well in triplicate into 12-well culture plates (Nunc, NY, USA) at passage 3. Cells were detached with 0.25% trypsin EDTA solution (Invitrogen) and counted every 2 days using a hemocytometer for 14 days.

To confirm the apoptosis rate of dDSSCs due to cryodamage, apoptotic cells were analyzed using a Dead Cell FITC Annexin V Apoptosis Detection Kit (Invitrogen). Briefly, dDSSCs in the fresh and cryogroups at passage 3 were washed with DPBS, resuspended in 100 *μ*l of 1× annexin-binding buffer, and stained with 5 *μ*l of Alexa Fluor annexin V and 1 *μ*l of 100 *μ*l/ml PI working solution (5 *μ*l of 1 mg/ml PI solution in 45 *μ*l 1× annexin-binding buffer) for 15 min at room temperature. Thereafter, cells were washed with DPBS and gently resuspended in 400 *μ*l 1× annexin-binding buffer. Cell viability was analyzed by measuring fluorescence emission at 530 and 575 nm upon excitation at 488 nm by flow cytometry. Cells were categorized as viable, early apoptotic, and late apoptotic. A total of 10,000 cells were acquired and analyzed.

### 2.7. Cell Surface Marker Analysis

Expression of positive (CD44, CD90) and negative (MHC II) surface markers of MSCs on dDSSCs in the fresh and cryogroups were analyzed by flow cytometry [28]. Cells at passage 3 were fixed with 4% formaldehyde solution at room temperature for 1 h.

After fixation, cells were stained with allophycocyanin- (APC-) conjugated anti-MHC II (monoclonal; BD Biosciences, Franklin Lakes, NJ, USA), fluorescein isothiocyanate- (FITC-) conjugated anti-CD44 (monoclonal, BD Biosciences), and APC-conjugated anti-CD90 (monoclonal, BD Biosciences) antibodies at 4°C for 1 h. A total of 1 × 10^4^ cells were assessed by flow cytometry. All antibodies were diluted 1 : 100 with 1% bovine serum albumin (BSA).

### 2.8. Real-Time Quantitative Polymerase Chain Reaction (RT-qPCR) Analysis

Expression of pluripotency and apoptosis-related genes was analyzed by real-time quantitative polymerase chain reaction (RT-qPCR). Total RNA was extracted from cells in the fresh and cryogroups using an easy-spin Total RNA Extraction Kit (iNtRON Biotechnology, Seongnam, Korea). The concentration of RNA was quantified using a Nanodrop 1000 spectrophotometer (Thermo Fisher Scientific). Complementary DNA (cDNA) was synthesized from 1 *μ*g of RNA using a HisenScript RT PreMix Kit (iNtRON Biotechnology) at 42°C for 1 h. We performed RT-qPCR using a Rotor-Gene Q cycler (Qiagen, Germantown, MD, USA) with RealMODTM Green AP 5 × qPCR mix (iNtRON Biotechnology) containing 50 ng of cDNA and 200 nM forward and reverse primers (Table 2). The RT-qPCR cycle was as follows: initial activation at 95°C for 12 min, followed by 40 cycles at 95°C for 15 s, 60°C for 25 s, and 72°C for 25 s. Gene expression was normalized to the mRNA level of the housekeeping gene, glyceraldehyde-3-phosphate Dehydrogenase (GAPDH). All samples were analyzed in triplicate.

### 2.9. Assessment of In Vitro Differentiation of dDSSCs into Adipocytes and Osteoblasts

dDSSCs in the fresh and cryogroups at passage 3 were induced to differentiate into adipocytes and osteoblasts as previously reported [20]. In brief, cells were cultured in suitable induction media. Adipogenic medium consisted of DMEM containing 10% FBS, 100 *μ*M indomethacin, 10 *μ*M insulin, and 1 *μ*M dexamethasone. Osteogenic medium consisted of DMEM containing 10% FBS, 10 nM dexamethasone, 50 *μ*g/ml ascorbic acid, and 10 mM sodium *β*-glycerophosphate. In vitro differentiation into adipocytes and osteoblasts was performed for 21 days, and media were changed every 2 days. Differentiated cells were fixed with 4% paraformaldehyde. After fixation, adipogenesis was confirmed by accumulation of lipid droplets detected by Oil Red O staining. Osteogenesis was confirmed by Alizarin Red S and von Kossa staining. Adipogenic and osteogenic differentiation was evaluated by RT-qPCR analysis of lineage-related genes (Table 2).

### 2.10. Mitochondrial Membrane Potential Analysis

The mitochondrial membrane potential was assessed using a JC-1 Mitochondrial Membrane Potential Assay Kit (Abnova, Taipei, Taiwan) according to the manufacturer's instructions. In brief, cells in the fresh and cryogroups were cultured in 6-well plates (Thermo Fisher Scientific) in DMEM supplemented with 10% FBS at 39°C in a humidified incubator containing 5% CO_2_ and 5% O_2_. Cells were washed twice with DPBS and treated with JC-1 staining solution at 39°C for 15 min. To label nuclei, cells were counterstained with 1 *μ*g/ml 4′,6-diamidino-2-phenylindole (DAPI) for 5 min. The mitochondrial membrane potential was evaluated as the ratio between JC-1 aggregates (high mitochondrial membrane potential) and monomers (low mitochondrial membrane potential).

### 2.11. Statistical Analysis

Data analyses were analyzed using SPSS for Windows (version 15, SPSS Inc., Chicago, IL, USA). Graphs were prepared with the GraphPad Prism (version 4.0) software. Statistical significance between mean values was assessed using Duncan's multiple range test. *p* values less than 0.05 were considered statistically significant.

## 3. Results

### 3.1. Histological Assessment and Survival Rates of Cells Derived from Fresh and Vitrified Dog Dermal Skin Tissues

**Figure 1:**
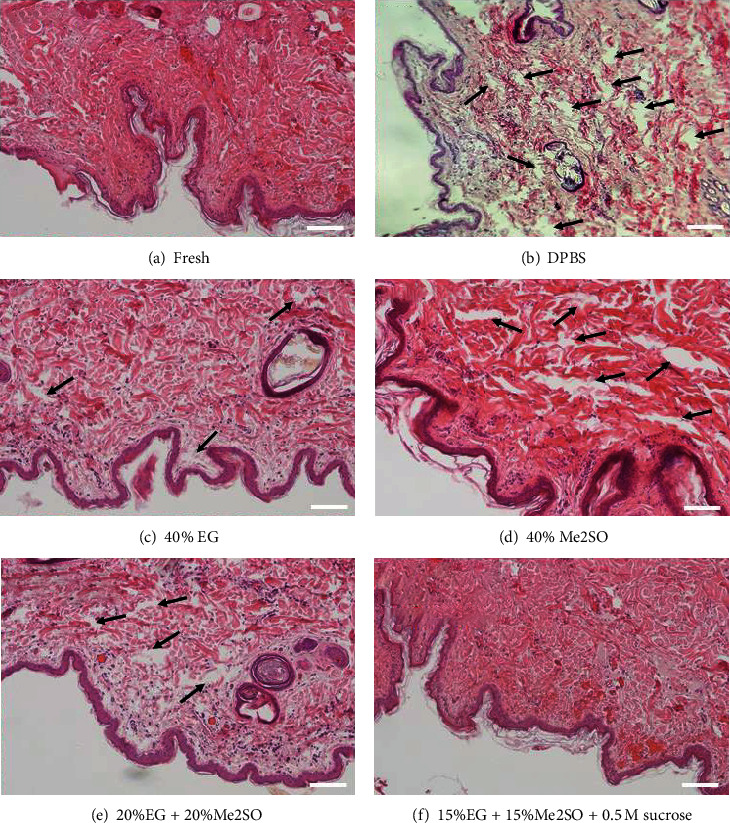
Histological assessment of dog skin tissues in the fresh and vitrification groups. Skin tissues were stained with H&E, and dense irregular connective tissues indicated that samples were normal: (a) fresh group; (b) DPBS group; (c) 40% EG group; (d) 40% Me2SO group; (e) 20%EG + 20%Me2SO group; (f) 15%EG + 15%Me2SO + 0.5 M sucrose group. Scale bar = 100 *μ*m. Black arrows indicate spaces due to freezing damage.

**Figure 2:**
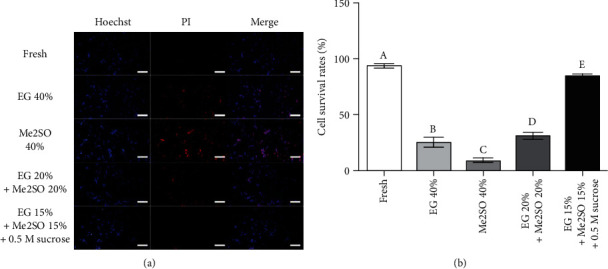
Survival rates of single cells isolated from dog skin tissues. (a) Isolated single cells in the fresh and vitrification groups were stained with Hoechst 33342 and propidium iodide (PI). Scale bar = 100 *μ*m. (b) Cell survival rates were calculated. The cell survival rate was highest in the fresh group and was significantly higher in the 15%EG + 15%Me2SO + 0.5 M sucrose group than in the other vitrification groups. Data represent the mean ± SD of four independent experiments. Subscript letters indicate significant differences between groups (*p* < 0.05).

We fixed fresh and vitrified dog skin tissues (DPBS, 40% EG, 40% Me2SO, 20%EG + 20%Me2SO, and 15%EG + 15%Me2SO + 0.5 M sucrose groups) and prepared paraffin sections. All tissues were stained with hematoxylin and eosin (H&E), and normal dense irregular connective tissues were observed in the fresh and 15%EG + 15%Me2SO + 0.5 M sucrose groups (Figure 1). Severe tissue damage was observed in the DPBS group. Furthermore, mild tissue damage was observed in the 40% EG, 40% Me2SO, and 20%EG + 20%Me2SO groups (Figure 1). The survival rates of dDSSCs were 94.1 ± 1.7%, 24.9 ± 4.2%, 8.6 ± 1.7%, 31.4 ± 3.1%, and 84.0 ± 2.3% in the fresh, 40% EG, 40% Me2SO, 20%EG + 20%Me2SO, and 15%EG + 15%Me2SO + 0.5 M sucrose groups, respectively (Figure 2). Cells were not established from the DPBS group. The cell survival rate was significantly (*p* < 0.05) higher in the fresh group than in the vitrification groups and was lowest in the 40% Me2SO group. However, the cell survival rate was significantly (*p* < 0.05) higher in the 15%EG + 15%Me2SO + 0.5 M sucrose group than in the other vitrification groups. Based on these results, tissues were vitrified using a cocktail of 15% EG, 15% Me2SO, and 0.5 M sucrose in subsequent experiments.

### 3.2. Morphology, Proliferation, Apoptosis, and Cell Surface Marker Expression of dDSSCs

**Figure 3:**
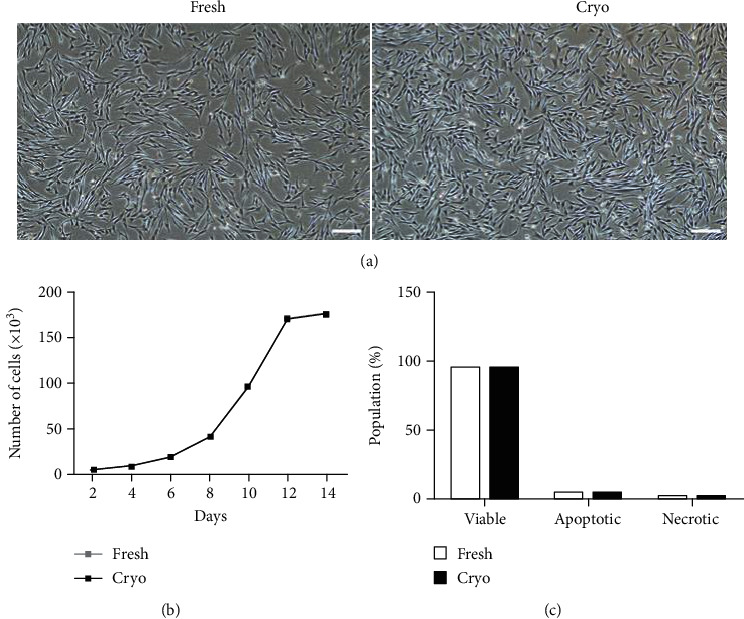
Morphology, growth characteristics, and apoptosis of dDSSCs in the fresh and cryogroups. (a) dDSSCs in the fresh and cryogroups had a spindle-like morphology. Scale bar = 200 *μ*m. (b) The proliferation capacity of dDSSCs in the fresh and cryogroups at passage 3 did not significantly differ. (c) The rate of apoptosis did not differ between dDSSCs in the fresh and cryogroups. Data represent by the mean ± SD of four independent experiments.

**Figure 4:**
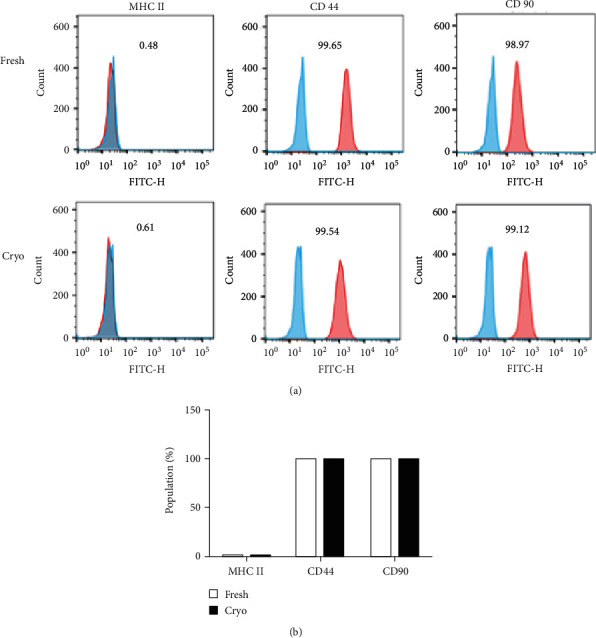
Expression of cell surface markers on dDSSCs in the fresh and cryogroups. (a, b) dDSSCs expressed mesenchymal markers (CD44 and CD90) and did not express MHC II in the fresh and cryogroups. Data represent by the mean ± SD of four independent experiments.

dDSSCs were isolated and cultured in the fresh and cryogroups. Adherent cells in both groups had a homogenous spindle-like morphology at passage 3 (Figure 3(a)). We analyzed cell viability and cellular apoptosis at passage 3. dDSSCs in the fresh and cryogroups had similar growth patterns (Figure 3(b)). Additionally, the cellular apoptosis was analyzed using the annexin V/PI assay. The percentages of viable, early apoptotic, and late apoptotic cells did not differ between the fresh and cryogroups (Figure 3(c)). The percentages of dDSSCs positive for MSC markers (CD44 and CD90) were similar in the fresh and cryogroups (Figure 4). However, almost no cells expressed MHC II (Figure 4).

### 3.3. Expression of Pluripotency and Apoptosis Markers

**Figure 5:**
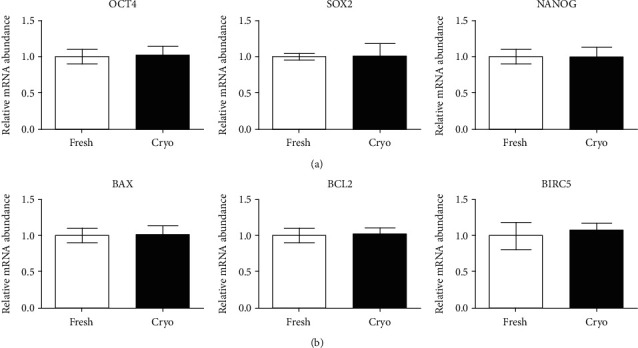
RT-qPCR analysis of dDSSCs in the fresh and cryogroups. (a, b) The mRNA levels of pluripotency (OCT4, SOX2, and NANOG), proapoptosis (BAX), and antiapoptotic (BCL2 and BIRC5) markers did not significantly differ between the groups. Data represent by the mean ± SD of four independent experiments.

We evaluated the expression levels of pluripotency and apoptosis markers. Total RNA was extracted from dDSSCs at passage 3 in the fresh and cryogroups. The expression levels of pluripotency markers (OCT4, SOX2, and NANOG) did not significantly differ between the two groups (Figure 5(a)). Furthermore, expression of proapoptotic (BAX) and antiapoptotic (BCL2 and BIRC5) markers was similar in the two groups (Figure 5(b)).

### 3.4. In Vitro Differentiation into Adipocytes and Osteoblasts

**Figure 6:**
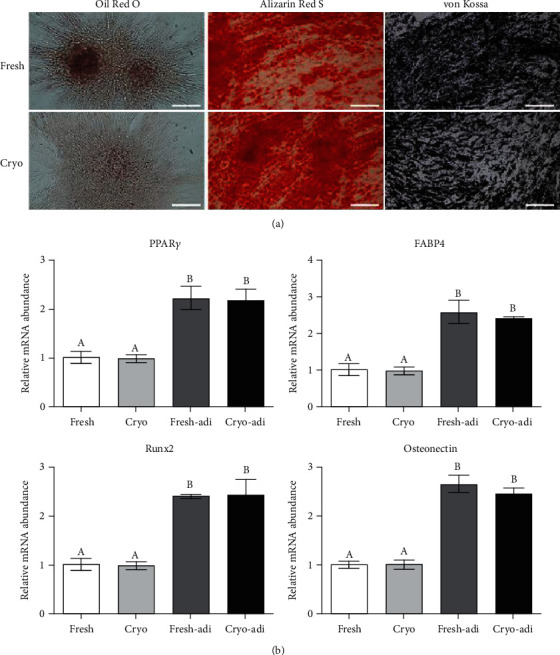
dDSSCs differentiated into adipocytes and osteoblasts in the fresh and cryogroups. (a) Cytochemical staining of induced adipocytes (Oil Red O) and osteoblasts (Alizarin Red S and von Kossa). (b) RT-qPCR analysis of gene expression of lineage-related markers in induced adipocytes and osteoblasts. There was no significant difference between the fresh and cryogroups. Data represent by the mean ± SD of four independent experiments.

dDSSCs in the fresh and cryogroups successfully differentiated into adipocytes and osteoblasts. Accumulation of lipid droplets was confirmed by Oil Red O staining, and mineral nodules were visualized by Alizarin Red S and von Kossa staining (Figure 6(a)). Furthermore, the mRNA levels of adipocyte- and osteoblast-specific genes were significantly (*p* < 0.05) higher in differentiated cells than in undifferentiated cells (Figure 6(b)). However, there was no difference between the fresh and cryogroups (Figure 6(b)).

### 3.5. Mitochondrial Membrane Potential Analysis

**Figure 7:**
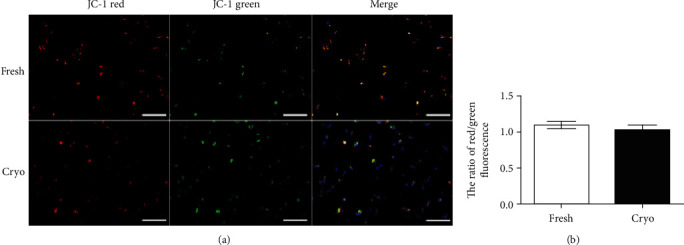
Mitochondrial membrane potential analysis of dDSSCs in the fresh and cryogroups. (a) JC-1 staining of dDSSCs in the fresh and cryogroups. Nuclei were stained with DAPI. Scale bar = 100 *μ*m. (b) The red/green (JC-1 aggregate/monomer) fluorescence ratio did not significantly differ between the two groups. Data represent by the mean ± SD of four independent experiments.

To assess whether vitrification induced mitochondrial damage in dDSSCs, the mitochondrial membrane potential was measured using a JC-1 Mitochondrial Membrane Potential Assay Kit. Red and green fluorescence indicates an increased and decreased mitochondrial membrane potential, respectively (Figure 7(a)). The red/green fluorescence ratio did not significantly differ between the fresh and cryogroups (Figure 7(b)).

## 4. Discussion

Skin tissue is a prominent source of MSCs for clinical applications. Although several studies have investigated cryopreservation of MSCs, vitrification of skin tissues has several advantages as mentioned in Introduction. Additionally, studies of MSCs derived from skin tissue after warming are insufficient. This study sought to develop a method for vitrification of dog skin tissue as a source of autologous stem cells for regenerative medicine. Vitrification methods have been reported using mammalian samples including oocytes, sperm, and tissues [29–31]. Several studies reported a high survival rate of follicles in vitrified tissue and successful pregnancy upon ovarian tissue transplantation [4, 32, 33]. Furthermore, studies of oocyte, testicular tissue, and ovarian tissue vitrification reported that use of intracellular and extracellular cryoprotectants reduces cytotoxicity [29–31]. Tissue vitrification is performed using a high concentration of cryoprotectant to prevent freezing and induce a glassy, vitrified state, and FBS, bovine serum albumin (BSA), and human albumin have been included to protect cells against cryoinjury and to increase the freezing efficiency [11, 17]. However, these xenogeneic factors can be a barrier to clinical applications. Therefore, we vitrified dog skin tissues using serum-free cryoprotectant cocktails. Several studies have successfully established MSCs by slow freezing of umbilical cord matrix and dental tissues [34–36]. However, limited studies have established stem cells and analyzed their characteristics using tissue vitrification. The present study modified ovarian tissue vitrification techniques for storage of dog skin tissues as a source of autologous stem cells.

As described, vitrification requires a high concentration of cryoprotectant. However, this can lead to problems including cytotoxicity and excessive cryoprotectant penetration of the cell membrane [14, 15]. To overcome these problems, studies have used cocktails containing various cryoprotectants. Me2SO has a slower permeation rate than EG, and the efficiency of embryo development lower using oocytes vitrified Me2SO than using oocytes vitrified with EG [37–39]. However, several recent studies reported that use of a cryoprotectant mixed with Me2SO, which has a slow permeation rate, and EG, which has a fast permeation rate, is effective for cryopreservation of oocytes and embryos [40, 41]. Furthermore, sugars including sucrose have a large molecular weight, facilitate dehydration of water during freezing, prevent swelling of the cytoplasm due to sudden changes in osmotic pressure during warming, and allow a lower concentration of an intracellular cryoprotectant to be used [11, 17]. Our results are consistent with previous studies. Histological analysis of fresh and vitrified tissues revealed that tissues were mildly damaged during vitrification and warming with a cryoprotectant cocktail (15%EG + 15%Me2SO + 0.5 M sucrose) (Figure 1). Additionally, the cell survival rate in the 15%EG + 15%Me2SO + 0.5 M sucrose group was lower than that in the fresh group but was significantly higher than that in the other vitrification group. No cells were established from tissues exposed to freezing without a cryoprotectant, which is likely due to severe tissue damage (Figure 1).

Stem cells lose their viability and proliferation capacity upon freezing and warming [12]. These changes hamper in clinical applications. dDSSCs were cultured to passage 3, and their characteristics, including proliferation and apoptosis, were evaluated. dDSSCs in the fresh and cryogroups had a spindle-like morphology. The proliferation capacity of dDSSCs did not differ between the two groups. We also analyzed apoptosis to confirm that dDSSCs recovered from cryodamage upon subculture. The percentages of viable, early apoptotic, and late apoptotic were similar in the fresh and cryogroups. Therefore, we speculated that cells derived from vitrified tissues were in a similar state to those derived from fresh tissues and conducted additional experiments.

Cell surface expression analysis revealed that dDSSCs in the fresh and cryogroups expressed CD44 and CD90 and did not express MHC II. Expression of pluripotency genes (OCT4, SOX2, and NANOG) was similar in the two groups. Furthermore, the mRNA expression levels of proapoptotic (BAX) and antiapoptotic (BCL2 and BIRC5) genes were similar in the fresh and cryogroups. These results are consistent with previous reports. Stem cells derived from tissues cryopreserved using the slow-freezing method express MSC-specific markers, and mRNA expression of pluripotency and apoptosis-related factors is similar in stem cells derived from cryopreserved and fresh tissues [4, 27]. Furthermore, cells in the fresh and cryogroups differentiated into adipocytes and osteoblasts, and their differentiation potentials were similar. The mitochondrial membrane potential is a crucial factor for ATP synthesis via oxidative phosphorylation [42]. Changes in the mitochondrial membrane potential affect cell viability and play an important role in mitochondrial homeostasis [42]. Our data showed that the ratio of red/green fluorescence upon JC-1 staining was similar in the fresh and cryogroups.

## 5. Conclusions

In conclusion, dDSSCs were successfully established using a cocktail of cryoprotectants, and their characteristics were analyzed. Cells derived from tissues vitrified using 15% EG, 15% Me2SO, and 0.5 M sucrose had a higher survival rate upon postwarming than cells in the other vitrification groups. The proliferation capacity, apoptosis rate, and stem cell characteristics, including expression of CD and pluripotency markers and potentials to undergo osteogenesis and adipogenesis, of dDSSCs derived from vitrified tissues (15% EG, 15% Me2SO, and 0.5 M sucrose) were similar to those of dDSSCs derived from fresh tissues. Furthermore, the mitochondrial membrane potential did not differ between the two groups. Although a further study is necessary to evaluate the effect of this vitrification method on the in vivo efficacy, the present study reports a technique that can be used as an alternative to the slow-freezing method and obtain autologous stem cells for clinical applications.

## Figures and Tables

**Table 1:** Summary of preequilibration and cryoprotectant solution in the present study.

Experimental group	Preequilibration solution	Cryoprotectant solution	Freezing method
Fresh (control)	No	No	No freezing
DPBS	DPBS	DPBS	Two steps^∗^
40% EG	20% EG in DPBS	40% EG in DPBS	Two steps^∗^
40% Me2SO	20% Me2SO in DPBS	40% Me2SO in DPBS	Two steps^∗^
20%EG + 20%Me2SO	10%EG + 10%Me2SO in DPBS	20%EG + 20%Me2SO in DPBS	Two steps^∗^
15%EG + 15%Me2SO + 0.5 M sucrose	7.5%EG + 7.5%Me2SO + 0.25 M sucrose in DPBS	15%EG + 15%Me2SO + 0.5 M sucrose in DPBS	Two steps^∗^

^∗^The vitrification process was conducted with two steps: Specifically, samples were incubated for 1 min at 25°C in preequilibration solution and then plunged into liquid nitrogen in cryoprotectant solution.

**Table 2:** Lists of primers used for RT-qPCR analysis.

Gene name (symbol)	Primer sequence	Product size (bp)	Annealing temperature (°C)
POU class 5 homeobox 1 (OCT4)	F: AACGATCAAGCAGTGACTATTCG	147	60
	R: AGTAGAGCGTAGTGAAGTGAGG		

Sex determining region Y-box 2 (SOX2)	F: AGTCTCCAAGCGACGAAAAA	189	60
	R: CCACGTTTGCAACTGTCCTA		

Nanog homeobox (NANOG)	F: GACCGTCTCTCCTCTTCCTTCC	157	60
	R: CGTCCTCATCTTCTGTTTCTTGC		

BCL2-associated X protein (BAX)	F: TTTGCTTCAGGGTTTCATCC	146	60
	R: TGTTACTGTCCAGTTCATCTCC		

B-cell lymphoma 2 (BCL2)	F: GGGTCATGTGTGTGGAGAGC	180	60
	R: GCCAGGAGAAGTCAAACAGAGG		

Baculoviral IAP repeat containing 5 (BIRC5)	F: ACATTCATCTGGTTGTGCTTTCC	157	60
	R: CACTTTCTTTGCGGTCTCTTCG		

Peroxisome proliferator-activated receptor gamma (PPAR*γ*)	F: GCAAGCACTTCACAAGAAACTACC	108	60
	R: ATGGGAGTGGTCATCCATTACG		

Fatty acid binding protein 4 (FABP4)	F: CTGGGCTCCAGAAGGTCATA	212	60
	R: GATGATCCTGTTGGGTTTGG		

Runt-related transcription factor 2 (Runx2)	F: TCTTCCCAAAGCCAGAGTGG	167	60
	R: TTTAATAGCGTGCTGCCATTCG		

Osteonectin	F: GGTGCTGAGGAAACTGAAGAGG	180	60
	R: CTTGTTGTCGTTGCTGCATACC		

Glyceraldehyde 3-phosphate dehydrogenase (GAPDH)	F: CCCATTCCATCTTCCAGAAA	196	60
	R: ATCTCACGGTTCCAGTTTGC		

## Data Availability

Data access can be requested on demand with the corresponding author.

## References

[1] Son Y. B., Kang Y. H., Lee H. J. (2021). Evaluation of odonto/osteogenic differentiation potential from different regions derived dental tissue stem cells and effect of 17*β*-estradiol on efficiency. *BMC Oral Health*.

[2] Park B. W., Kang D. H., Kang E. J. (2012). Peripheral nerve regeneration using autologous porcine skin-derived mesenchymal stem cells. *Journal of Tissue Engineering and Regenerative Medicine*.

[3] Park H. C., Son Y. B., Lee S. L. (2017). Effects of osteogenic-conditioned medium from human periosteum-derived cells on osteoclast differentiation. *International Journal of Medical Sciences*.

[4] Chen S. U., Chien C. L., Wu M. Y. (2006). Novel direct cover vitrification for cryopreservation of ovarian tissues increases follicle viability and pregnancy capability in mice. *Human Reproduction*.

[5] Moore K., Bonilla A. Q. (2007). Cryopreservation of mammalian embryos: the state of the Art. *Annual Review of Biomedical Sciences*.

[6] Leonel E. C. R., Corral A., Risco R. (2019). Stepped vitrification technique for human ovarian tissue cryopreservation. *Scientific Reports*.

[7] Isachenko V., Lapidus I., Isachenko E. (2009). Human ovarian tissue vitrification versus conventional freezing: morphological, endocrinological, and molecular biological evaluation. *Reproduction*.

[8] Kelly S. M., Buckett W. M., Abdul-Jalil A. K., Tan S. L. (2003). The cryobiology of assisted reproduction. *Minerva Ginecologica*.

[9] Arav A., Yavin S., Zeron Y., Natan D., Dekel I., Gacitua H. (2002). New trends in gamete's cryopreservation. *Molecular and Cellular Endocrinology*.

[10] Mathias F. J., D’Souza F., Uppangala S., Salian S. R., Kalthur G., Adiga S. K. (2014). Ovarian tissue vitrification is more efficient than slow freezing in protecting oocyte and granulosa cell DNA integrity. *Systems Biology in Reproductive Medicine*.

[11] Liebermann J., Nawroth F., Isachenko V., Isachenko E., Rahimi G., Tucker M. J. (2002). Potential importance of vitrification in reproductive medicine. *Biology of Reproduction*.

[12] Amorim C. A., Curaba M., van Langendonckt A., Dolmans M. M., Donnez J. (2011). Vitrification as an alternative means of cryopreserving ovarian tissue. *Reproductive Biomedicine Online*.

[13] Wusteman M. C., Pegg D. E., Wang L., Robinson M. P. (2003). Vitrification of ECV304 cell suspensions using solutions containing propane-1,2-diol and trehalose. *Cryobiology*.

[14] Li Y., Tan J. C., Li L. S. (2010). Comparison of three methods for cryopreservation of human embryonic stem cells. *Fertility and Sterility*.

[15] Vanderzwalmen P., Zech N., Lejeune B., Wirtleitner B., Zech M., Ectors F. (2010). Vitrification and the use of high concentrations of cryoprotectants: is it a justified argument to prefer slow freezing?. *Gynécologie, Obstétrique & Fertilité*.

[16] Bautista J. A., Kanagawa H. (1998). Current status of vitrification of embryos and oocytes in domestic animals: ethylene glycol as an emerging cryoprotectant of choice. *The Japanese Journal of Veterinary Research*.

[17] David E. P. (2005). The role of vitrification techniques of cryopreservation in reproductive medicine. *Human Fertility (Cambridge, England)*.

[18] Varma V. P., Devi L., Venna N. K., Murthy C. L. N., Idris M. M., Goel S. (2015). Ocular fluid as a replacement for serum in cell cryopreservation media. *PLoS One*.

[19] Latinwo L., Badisa V., Odewumi C. (2008). Comparative evaluation of cytotoxicity of cadmium in rat liver cells cultured in serum-containing medium and commercially available serum-free medium. *International Journal of Molecular Medicine*.

[20] Shivakumar S. B., Bharti D., Subbarao R. B. (2016). DMSO- and serum-free cryopreservation of Wharton’s jelly tissue isolated from human umbilical cord. *Journal of Cellular Biochemistry*.

[21] Taylor M. J., Weegman B. P., Baicu S. C., Giwa S. E. (2019). New approaches to cryopreservation of cells, tissues, and organs. *Transfusion Medicine and Hemotherapy*.

[22] Silvestre M. A., Saeed A. M., Escribá M. J., García-Ximénez F. (2002). Vitrification and rapid freezing of rabbit fetal tissues and skin samples from rabbits and pigs. *Theriogenology*.

[23] Silvestre M. A., Sánchez J. P., Gómez E. A. (2004). Vitrification of goat, sheep, and cattle skin samples from whole ear extirpated after death and maintained at different storage times and temperatures. *Cryobiology*.

[24] Borges A. A., Lira G. P. O., Nascimento L. E. (2018). Influence of cryopreservation solution on the in vitro culture of skin tissues derived from collared peccary (Pecari tajacu Linnaeus, 1758). *Biopreservation and Biobanking*.

[25] Santos R. R., Tharasanit T., van Haeften T., Figueiredo J. R., Silva J. R. V., van den Hurk R. (2007). Vitrification of goat preantral follicles enclosed in ovarian tissue by using conventional and solid-surface vitrification methods. *Cell and Tissue Research*.

[26] Wang Y., Xiao Z., Li L., Fan W., Li S. W. (2008). Novel needle immersed vitrification: a practical and convenient method with potential advantages in mouse and human ovarian tissue cryopreservation. *Human Reproduction*.

[27] Park B. W., Jang S. J., Byun J. H. (2017). Cryopreservation of human dental follicle tissue for use as a resource of autologous mesenchymal stem cells. *Journal of Tissue Engineering and Regenerative Medicine*.

[28] Russell K. A., Chow N. H. C., Dukoff D. (2016). Characterization and immunomodulatory effects of canine adipose tissue- and bone marrow-derived mesenchymal stromal cells. *PLoS One*.

[29] Andrae C. A., Oliveira E. C. S., Ferraz M. A. M. M., Nagashima J. B. (2021). Cryopreservation of grey wolf (*Canis lupus*) testicular tissue. *Cryobiology*.

[30] Gallardo M., Saenz J., Risco R. (2019). Human oocytes and zygotes are ready for ultra-fast vitrification after 2 minutes of exposure to standard CPA solutions. *Scientific Reports*.

[31] Tao Y., Sanger E., Saewu A., Leveille M. C. (2020). Human sperm vitrification: the state of the art. *Reproductive Biology and Endocrinology*.

[32] Courbiere B., Massardier J., Salle B., Mazoyer C., Guerin J. F., Lornage J. (2005). Follicular viability and histological assessment after cryopreservation of whole sheep ovaries with vascular pedicle by vitrification. *Fertility and Sterility*.

[33] Kagawa N., Silber S., Kuwayama M. (2009). Successful vitrification of bovine and human ovarian tissue. *Reproductive Biomedicine Online*.

[34] Chen Y. K., Huang A. H. C., Chan A. W. S., Shieh T. Y., Lin L. M. (2011). Human dental pulp stem cells derived from different cryopreservation methods of human dental pulp tissues of diseased teeth. *Journal of Oral Pathology & Medicine*.

[35] Ma L., Makino Y., Yamaza H. (2012). Cryopreserved dental pulp tissues of exfoliated deciduous teeth is a feasible stem cell resource for regenerative medicine. *PLoS One*.

[36] Seo B. M., Miura M., Sonoyama W., Coppe C., Stanyon R., Shi S. (2005). Recovery of stem cells from cryopreserved periodontal ligament. *Journal of Dental Research*.

[37] Wallach E. E., Friedler S., Giudice L. C., Lamb E. J. (1988). Cryopreservation of embryos and ova. *Fertility and Sterility*.

[38] Martino A., Songsasen N., Leibo S. P. (1996). Development into blastocysts of bovine oocytes cryopreserved by ultra-rapid cooling. *Biology of Reproduction*.

[39] Sommerfeld V., Niemann H. (1999). Cryopreservation of bovine in vitro produced embryos using ethylene glycol in controlled freezing or vitrification. *Cryobiology*.

[40] Huang L., Mo Y., Wang W., Li Y., Zhang Q., Yang D. (2008). Cryopreservation of human ovarian tissue by solid-surface vitrification. *European Journal of Obstetrics, Gynecology, and Reproductive Biology*.

[41] Lucena E., Bernal D. P., Lucena C., Rojas A., Moran A., Lucena A. (2006). Successful ongoing pregnancies after vitrification of oocytes. *Fertility and Sterility*.

[42] Zorova L. D., Popkov V. A., Plotnikov E. Y. (2018). Mitochondrial membrane potential. *Analytical Biochemistry*.

